# Molecular basis for prey relocation in viperid snakes

**DOI:** 10.1186/1741-7007-11-20

**Published:** 2013-03-01

**Authors:** Anthony J Saviola, David Chiszar, Chardelle Busch, Stephen P Mackessy

**Affiliations:** 1 School of Biological Sciences, University of Northern Colorado, 501 20th St., CB 92, Greeley, CO 80639-0017 USA; 2 Department of Psychology, University of Colorado at Boulder, CB 345, Boulder, CO 80309 USA

**Keywords:** *Crotalus*, disintegrin, evolution, phenotype, mass spectrometry, predation, protein sequence, toxin, venom

## Abstract

**Background:**

Vertebrate predators use a broad arsenal of behaviors and weaponry for overcoming fractious and potentially dangerous prey. A unique array of predatory strategies occur among snakes, ranging from mechanical modes of constriction and jaw-holding in non-venomous snakes, to a chemical means, venom, for quickly dispatching prey. However, even among venomous snakes, different prey handling strategies are utilized, varying from the strike-and-hold behaviors exhibited by highly toxic elapid snakes to the rapid strike-and-release envenomation seen in viperid snakes. For vipers, this mode of envenomation represents a minimal risk predatory strategy by permitting little contact with or retaliation from prey, but it adds the additional task of relocating envenomated prey which has wandered from the attack site. This task is further confounded by trails of other unstruck conspecific or heterospecific prey. Despite decades of behavioral study, researchers still do not know the molecular mechanism which allows for prey relocation.

**Results:**

During behavioral discrimination trials (vomeronasal responsiveness) to euthanized mice injected with size-fractionated venom, *Crotalus atrox *responded significantly to only one protein peak. Assays for enzymes common in rattlesnake venoms, such as exonuclease, L-amino acid oxidase, metalloproteinase, thrombin-like and kallikrein-like serine proteases and phospholipase A_2_, showed that vomeronasal responsiveness was not dependent on enzymatic activity. Using mass spectrometry and N-terminal sequencing, we identified the proteins responsible for envenomated prey discrimination as the non-enzymatic disintegrins crotatroxin 1 and 2. Our results demonstrate a novel and critical biological role for venom disintegrins far beyond their well-established role in disruption of cell-cell and cell-extracellular matrix interactions.

**Conclusions:**

These findings reveal the evolutionary significance of free disintegrins in venoms as the molecular mechanism in vipers allowing for effective relocation of envenomated prey. The presence of free disintegrins in turn has led to evolution of a major behavioral adaptation (strike-and-release), characteristic of only rattlesnakes and other vipers, which exploits and refines the efficiency of a pre-existing chemical means of predation and a highly sensitive olfaction system. This system of a predator chemically tagging prey represents a novel trend in the coevolution of predator-prey relationships.

## Background

Coevolution within predator-prey interactions has led to adaptations that are advantageous for either prey capture or predation avoidance. In predators, these traits may be under strong selection leading to successful capture of prey [1,2], but they are relatively under-studied compared to the mechanisms involved in anti-predator adaptations [3]. Darwin [4] suggested that diversification of predators may be largely based on selection on predatory behaviors, and adaptations to observable phenotypic characteristics that are advantageous to prey capture are commonly examined. For example, evolution of craniofacial asymmetries has shown to increase predation success in scale-eating cichlids [5] as well as in snail-eating snakes [6]. Phenotypic plasticity undoubtedly plays a critical role in diversification of predators and prey, often leading to adaptations in behavior, life history, physiology and morphology of species [7]. Further, competition, predation and utilization of dangerous prey have been proposed as the most significant factors of selection on organisms [8]. The ability of predators to adapt to dangerous prey, such as garter snake (*Thamnophis sirtalis*) resistance to tetrodotoxin (TTX) of *Taricha *newts [2], provides strong evidence for a coevolutionary arms race between predators and prey. However, adaptations in predatory behaviors to avoid complete retaliation from dangerous prey may be rare. Nevertheless, natural selection can be expected to lead to adaptations influencing behaviors that are most advantageous to prey capture [1], and further examination of the molecular mechanisms allowing for these large scale behavioral adaptations is critical for understanding coevolution between predator-prey interactions. Many studies examining phenotypic plasticity in species address various forms of plasticity separately, yet this variety may have significantly different ecological consequences [9]. Among venomous snakes, venom characteristics are under positive directional selection [10], and the presence of specific venom components may have played a critical role in diversification of predatory behaviors of several snake taxa.

Rattlesnakes and other vipers demonstrate one of the most advanced modes of predation among vertebrates, utilizing a strike-and-release mode of envenomation. This behavior provides the benefit of minimal contact or retaliation from potentially dangerous prey, but adds the additional task of locating the trail left behind by the envenomated prey that may wander several meters or more from the attack site. By using rapid tongue flicking (strike-induced chemosensory searching) to detect, and the vomeronasal organs to analyze volatile and non-volatile chemical cues [11], snakes must then differentiate between the trail deposited by the prey before and after envenomation has occurred, as well as the trails left inadvertently by other potential prey and non-prey sources. Several hypotheses have addressed the source of chemical cues used to discriminate between trails of struck and unstruck prey. Cues emanating from the mouse when it is punctured during the envenomating strike, as well as other potential chemical cues, such as urine or volatiles from venom left on the prey's integument, have been examined, yet are not utilized by snakes [12-15]. These previous results indicate that venom must be injected into tissues to initiate a release of chemical odor(s), permitting discrimination of envenomated prey and their trails. A convenient bioassay of vomeronasal chemoreception was previously developed for evaluating preference towards envenomated (E) vs. non-envenomated (NE) mouse carcasses, with snakes showing high rates of tongue flicking directed toward E carcasses (strike-induced chemosensory searching, SICS [15-18]). This preference holds when envenomation occurs by a conspecific or by a closely related heterospecific [17], or when lyophilized conspecific venom is injected into previously euthanized prey [18]. Therefore, venoms represent not only a rapid-acting chemical means of dispatching potentially fractious prey [19]; they also greatly increase the perceptibility of the envenomated prey carcass [15,18]. However, the specific component(s) of snake venom allowing for successful recovery of prey and further diversification of prey handling behaviors has not been identified.

## Results

To determine which component(s) of venom allows for rattlesnakes to differentiate between envenomated (E) and non-envenomated (NE) prey, we offered western diamondback rattlesnakes (*Crotalus atrox*) E and NE mouse carcasses; E mice were injected with either crude venom or with fractionated protein or peptide peaks of crude venom (extracted from conspecifics). Non-envenomated mice were injected with a saline control. When artificial envenomation occurred with whole crude venom, the mean number of tongue flicks was significantly greater for the E mouse (*t *= 3.67, df = 6, *P *< 0.01; Table 1; see also Additional file 1, Table S1). When total number of tongue flicks were converted to percentage of tongue flicks (to control for natural variation in absolute tongue flick rate between snakes), results confirmed that *C. atrox *directed significantly more tongue flicks at the E than at NE mice (*t *= 3.76, df = 6, *P *< 0.01) (Table 1). These results agree with numerous studies of vomeronasal response of rattlesnakes to E versus NE prey [15,17,18], including a previous study performed using the same pool of *C. atrox *venom as used in this report [18].

**Table 1:** Rattlesnakes discriminate between non-envenomated and envenomated mice

Sample	NE	E	*t*
Venom (n = 7)	32 (8.45)	83 (15.9)	3.67**
	29	71 (5.65)	3.76**

Mean number of tongue flicks and mean percent (lower values) tongue flicks (s.e.m.) directed at non-envenomated (NE) and envenomated (E) mice by *Crotalus atrox *when mice were envenomated by whole crude venom. Single-sample *t*-test was conducted on mean percentages where mean percent to E mice were compared with 50%, the value expected under the null hypothesis; df = 6. Because the two means are not independent, the same *t *value but with the opposite sign would be obtained for each mean. For raw data, see Additional file 1, Table S1. ** *P *< 0.01.

**Figure 1:**
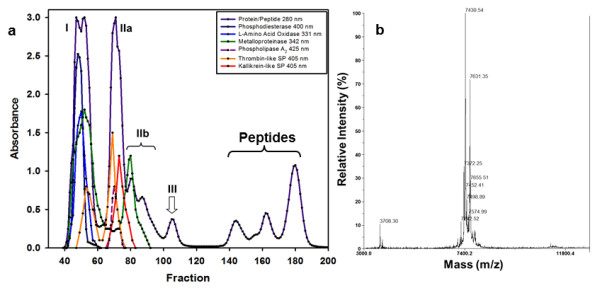
**Discrimination of envenomated prey is not dependent on enzymatic toxins**. **(A) **Size exclusion fractionation of 250 mg crude *C. atrox *venom on a 90 × 2.8 cm BioGel P-100 column equilibrated with HEPES/NaCl/CaCl_2 _buffer. Fractionation occurred at a flow rate of 6.3 mL per hour at 4°C, and eluting proteins/peptides were followed by absorbance at 280 nm. Enzyme activities common to rattlesnake venoms were assayed and are limited to the first two peaks. Arrow indicates the peak containing crotatroxins 1 and 2 (Peak III). **(B) **MALDI-TOF-MS analysis of peptides in BioGel size exclusion Peak III. Approximately 0.5 μg protein was spotted onto sinapinic acid matrix and analyzed using a mass window of 3 to 25 kD. Several peptides with masses typical of monomeric disintegrins (7,245 to 7,655 Da) were present, but no larger proteins were observed.

To test snake responses toward fractionated protein and peptide peaks, crude *C. atrox *venom was separated using low-pressure size exclusion liquid chromatography, and four major protein peaks, labeled I, IIa, IIb and III, as well as three downstream peptide peaks, were resolved (Figure 1A). When mouse carcasses were envenomated with either Peaks I, IIa, IIb or the peptide peaks, there was no significant difference between the mean number of tongue flicks or the percentages of tongue flicks directed towards either the E or NE carcasses (Table 2; see also Additional file 1, Table S1). However, for Peak III, there were significantly more tongue flicks directed towards the E mouse (*t *= 4.24, df = 10, *P *< 0.01; Table 2), and the mean percentage of tongue flicks toward the envenomated carcass (68%) was also significantly higher than the null (*t *= 5.78, df = 10, *P *< 0.01; Table 2). Analysis of variance (ANOVA) indicated a significant main effect of conditions (*F *= 4.63, df = 4, 54, *P *< 0.01). The Newman-Kewls range test also revealed that the mean for Peak III was significantly higher than the means for Peaks I, IIa, IIb and the peptide peaks (*P *< 0.05), which did not differ significantly among themselves (*P *> 0.05). Further, in 10 out of 11 Peak III trials, snakes tongue flicked more towards the E mouse (χ^2 ^= 3.68, df = 1, *P *= 0.05), whereas for Peaks I, IIa, IIb and the combined peptide peaks, there was no preference shown over the E mouse or the NE mouse (χ^2 ^= 0.264, 0.045, 0.2 and 0.05, respectively; all df's = 1, all *Ps *> 0.05).

**Table 2:** Prey discrimination is associated with non-enzymatic fractions.

Fraction	NE	E	*t*	df
Peak I	67.4 (11.9) 51	68.7 (12.3) 49 (5.3)	0.09 0.22	16
Peak IIa	72.9 (15.6) 50	59.0 (12.7) 50 (7.0)	0.70 0.01	10
Peak IIb	73.3 (18.4) 49	69.2 (15.3) 51 (4.3)	0.22 0.25	9
Peak III	25.3 (5.1) 32	53.6 (7.7) 68 (3.2)	4.24** 5.78**	10
Small peptide peaks (combined)	33.0 (7.7) 49	52.7 (24.6) 51 (11.7)	0.79 0.12	8

Mean number of tongue flicks and mean percent (lower values) tongue flicks (s.e.m.) directed at non-envenomated (NE) and envenomated (E) mice by *Crotalus atrox *when mice were envenomated using BioGel Peaks I, IIa, IIb, III or combined peptide peaks. Single-sample *t*-test was conducted on mean percentages in which mean percent tongue flicks to E mice were compared with 50%, the value expected under the null hypothesis. Because the two means within each paired comparison are not independent, the same *t-*value but with the opposite sign would be obtained for each mean. For raw data, see Additional file 1, Table S1. ** *P *< 0.01.

**Figure 2:**
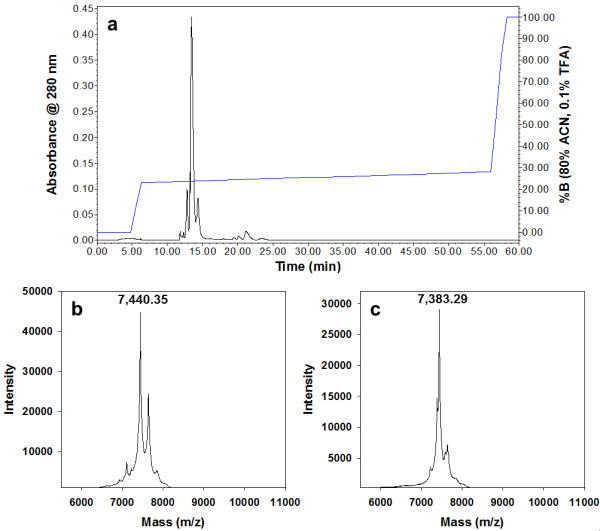
**Peak III consist only of 7 kDa peptides**. **(A) **Reversed-phase chromatography of Peak III from the gel filtration step (BioGel P-100). Two hundred microliters was injected onto a Vydac C_18 _(4.6 × 250 mm) column, and disintegrin peaks were eluted at 23% buffer B (13 to 14 minutes). **(B) **MALDI-TOF-MS analysis of crotatroxin 1 from the reverse-phase chromatography purification step (fraction 13). Mass of 7,440.35 was observed for crotatroxin 1. **(C) **MALDI-TOF-MS analysis of crotatroxin 2 from the reverse-phase chromatography purification step (fraction 14). Mass of 7,383.29 was observed for crotatroxin 2.

**Figure 3:**
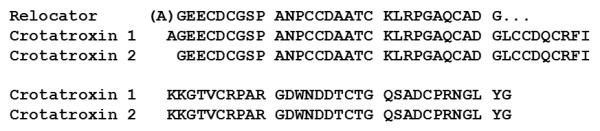
**N-terminal sequence of Peak III peptides (Relocator) confirms identity with crotatroxins (CT) 1 and 2**. Note that CTs 1 and 2 are identical in sequence except for the additional N-terminal alanine residue in CT1. Protein sequencing of the relocator peak showed lower yield (approximately 3 pmol, compared to approximately 6.5 pmol for residues 2 to 6) and presence of an N-terminal alanine at residue 1, indicating that both CTs were present. No secondary sequence (indicative of potential contaminant proteins) was observed.

We next sought to examine the components in Peak III that produced this significant vomeronasal response. Because metalloproteinase enzymes are prevalent components of most viper venoms [20] and because they would still catalyze degradation of non-living E mouse tissues, we hypothesized that these enzymes would be responsible for "tagging" of E prey. Assays for enzymes common in rattlesnake venoms (exonuclease, L-amino acid oxidase, metalloproteinase, thrombin-like and kallikrein-like serine proteases, and phospholipase A_2_: [21]) indicated that all of these activities were confined to Peaks I through IIb (Figure 1A). SDS-PAGE (Additional file 2, Figure S1) and mass spectrometry of Peak III (Figure 1B) revealed only peptides with masses of approximately 7.5 kD. Further analysis of Peak III through reverse-phase high pressure liquid chromatography (HPLC) yielded two peaks (Figure 2A) that were subjected to Matrix Assisted Laser Desorption Ionization Time-of-Flight (MALDI-TOF) mass spectrometer analysis. These results yielded masses of 7,440.35 Da (Figure 2B) and 7,383.29 Da (Figure 2C), respectively, indicating that the proteins isolated were the disintegrins crotatroxin 1 and crotatroxin 2. N-terminal sequencing of Peak III proteins confirmed the identity of these disintegrins (Figure 3).

## Discussion

Determining the molecular mechanisms leading to large-scale adaptations of predatory behaviors, including, in this case, relocation of prey, is critical for understanding predator-prey interactions, evolutionary biology and natural history of pit vipers. Our findings show that the venom disintegrins crotatroxin 1 and 2 alone allowed *C. atrox *to distinguish between envenomated and non-envenomated prey sources, presumably by altering the chemical odor of prey integument. Crotatroxins are medium-sized monomeric disintegrins with approximate masses of 7.4 kDa and contain 71 to 72 amino acids with six disulfide bonds, differing only by the presence of an additional N-terminal alanine in crotatroxin 1 ([22]; see also Figure 3). Disintegrins are non-enzymatic and are produced by the proteolytic posttranslational processing of the C-terminal domain of P-II snake venom metalloproteinases [23]. The presence of dimeric disintegrins in other viperid venoms has also been documented; however, only medium-sized monomeric disintegrins appear to be present in *C. atrox *venom [24]. It is currently unknown if dimeric disintegrins will produce the same type of vomeronasal response as the monomeric disintegrins did in this current study. A primary activity of disintegrins is the inhibition of platelet aggregation by selectively binding integrin receptors expressed on cell surfaces [25]. The majority of monomeric disintegrins, including crotatroxins 1 and 2, contain an active Arg-Gly-Asp (RGD) sequence [26], which has been shown to block numerous classes of integrin receptors with a high degree of selectivity. Therefore, the action of crotatroxins which results in successful relocation of envenomated prey via SICS likely involves an integrin binding mechanism and further release of volatile cues detectable by rattlesnakes.

Rattlesnake venoms are classified as either type I venoms, containing high metalloproteinase activity and lower toxicity, or type II venoms, containing low metalloproteinase activity and higher toxicity [21]. Although some strike-and-release rattlesnakes, such as *C. scutulatus scutulatus *(type A) and *C. tigris*, contain less than 0.1% venom metalloproteinases, proteomic studies have identified disintegrins in their venoms [27,28]. These species possess type II venoms with potent lethal toxicity, so the possibility of prey wandering a significant distance from the attack site before it has succumbed to venom is much less likely than species exhibiting type I venom, making relocation following a strike less challenging for these highly toxic rattlesnakes. Disintegrins make up approximately 2% (by mass) of the total venom proteins/peptides of crude *C. atrox *venom, though the abundance of this protein (and other venom compounds) may vary between individual snakes. The utilization of a relatively minor venom component to "tag" envenomated prey may also explain the "overkill method" [29] employed by venomous snakes. It has long been observed that many taxa of venomous snakes inject prey with amounts of venom which vastly exceed the mouse model LD_50_, often by several orders of magnitude [14,30]. In part, this "excessive" dosage is explained by differential sensitivity of various prey to specific toxins [31] and venoms [32], induced by coevolutionary responses of both prey and their snake predators [33]. For example, some prey species are much less affected by venoms, while others are highly sensitive (cf. frogs and lizards [34]). However, another important factor, in particular, among the strike-and-release predators, such as most viperids, is the need to discriminate between competing prey trails (E and NE rodents), selecting the one leading to the previously envenomated prey. This is likely a main reason why rattlesnakes use apparently large quantities of venom - to achieve a "minimum perceptible dose" [18].

Venoms consist of a myriad of proteins and peptides that may vary based on age, geographic locations and prey preference of the snake [35] . This complexity of venom composition, coupled with the fact that many species specialize on specific prey, likely result in selective pressures on venom characteristics, leading to the evolution of advantageous venom phenotypes and predatory behaviors [36]. On a trophic level, the roles of disintegrins and many other proteins found in venoms still remain relatively unknown. In whole venom, disintegrins which have not been proteolytically processed could potentially assist in the targeting of PII snake venom metalloproteinases (SVMPs) to specific integrin receptors in cell membranes [37], giving rise to chemical changes recognized by the snakes. Lys49 phospholipase A_2_s have also been suggested to act as a tag of envenomated prey [38]; however, we have demonstrated that neither the metalloproteinase-containing nor the PLA_2_-containing fractions of *C. atrox *venom elicited prey relocating responses.

To the best of our knowledge, all pit vipers that have been tested have shown significant preference for envenomated prey [for example, 15-18], indicating that disintegrins in other venoms, not just those in *C. atrox*, assist in prey relocation for other pit viper species. But not all snake venoms contain disintegrins. How are prey relocated in these cases? *Atractaspis *species (mole "vipers") use a unilateral slashing envenomation behavior to feed on neonatal rodents within nests and burrows [39], and prey escape after envenomation is highly improbable. Elapids are typically strike-and-hold predators [40], with venoms rich in rapid-acting three-finger toxins [35], and so the presence of a "relocator protein" in these venoms is not likely advantageous. Similarly, neonate rattlesnakes that generally strike-and-hold prey [41] produce much smaller amounts of venom and have significantly lower concentrations of metalloproteinases, the protein family that releases free disintegrins, when compared to venoms of subadult and adult rattlesnakes [21,41]. A major selective advantage for the evolution of free disintegrins among viperid venoms (apparently exclusively) is provided by their role in prey relocation.

Natural selection undoubtedly has influenced snake responses to stimuli that are most likely to lead to successful capture or, as in this case, successful relocation of prey [1,42]. Further, this preference for envenomated prey is an adaptive mechanism that facilitates optimal foraging efforts, leading to rapid relocation of prey after it has succumbed. Snakes often will not attend to a second prey offered after the initial envenomating strike, suggesting that chemical cues arising from the struck prey may be focusing foraging efforts and redirecting the snake from additional, potentially confounding chemical cue sources [43]. Our results strongly indicate that for *C. atrox*, disintegrins have evolved into multifunctional proteins which evoke vomeronasally-salient cues, enabling the snake to relocate envenomated prey after the strike. Therefore, in addition to immobilizing, killing and predigesting prey, another biological role of venoms in rattlesnakes is for prey relocation.

## Conclusions

These findings provide an important biological role for a non-lethal venom protein which has little apparent relevance to the well-characterized roles of disintegrins in disrupting cell-cell and cell-extracellular matrix interactions. Thus, in order to understand the evolution of animal venoms and venom compositional variation, it will be important to consider possible selective advantages conferred by specific venom components to the behavior and ecology of the animals which produce them, in addition to the more apparent pharmacological effects. At present, it is unknown how the crotatroxins create an olfactory "mark" that snakes are able to recognize, but we hypothesize that integrin-mediated release of chemical cues from prey stimulate the vomeronasal system of snakes. Studies now in progress are aimed at determining the mechanism(s) by which disintegrins interact with prey tissues and facilitate relocation of envenomated prey by rattlesnakes.

## Methods

### Materials

BioGel P-100 resin was obtained from BioRad, Inc. (San Diego, CA, USA). Matrix for MALDI-TOF-MS, enzyme substrates, buffer salts and all other reagents were analytical grade or better and were obtained from Sigma Chemical Co. (St. Louis, MO, USA).

### Experimental animals

Behavioral trials were performed as approved by the Institutional Animal Care and Use Committee of the University of Colorado at Boulder. Eight *C. atrox*, all adult long-term captive snakes, were fed bi-weekly on live or pre-killed mice (*Mus musculus*). Snakes were never fed on the day of trials, which occurred 7 to 10 days after the last feeding session, and all trials were randomized and separated by at least 14 days. Snakes were housed individually in glass aquaria (61.0 × 41.0 × 44.5 cm) containing a paper floor, water bowls and hide boxes. We maintained the snakes on a 12:12 L:D cycle and at 26 ± 2°C. Inbred Swiss/Webster mice (*Mus musculus*) were culls from colonies maintained by the University of Colorado Department of Molecular, Cellular and Developmental Biology and were euthanized by CO_2 _asphyxiation and frozen at -20° until used in this study [17]. The magnitude of SICS towards natural rodent prey such as *Peromyscus maniculatus *(deer mice) does not differ compared to lab mice (*M. musculus*) [44], and the strain of lab mice used also does not influence results. On testing days, similar size and sex mice were thawed and warmed by electrical heaters until skin temperature was 38 ± 1°C before injection and subsequent testing.

### Experiment 1

Venoms were manually extracted, centrifuged to pellet insoluble material, frozen, lyophilized and stored at -20°C until used [41]. Lyophilized venom was reconstituted on the day of testing by dissolving 10 mg of crude venom in 100 μL of deionized water. During a test day, *C. atrox *were allowed to strike and envenomate prey carcasses suspended from long forceps to initiate strike-induced chemosensory searching [15]. Since rattlesnakes release prey after the strike, this envenomated mouse was removed from the snakes' cages and discarded, and that mouse never touched the floor or walls of the cage. The test apparatus, a 4 × 10 cm metal base with two wire mesh baskets approximately 4.0 cm apart, containing both an envenomated mouse injected with 100 μL of reconstituted venom and a non-envenomated mouse [17,18], was placed into the snake's cage. The 100 μL volume of reconstituted venom is comparable to the volume of venom injected during a predatory strike [14]. Two injections (each containing 50 μL) were made in the thoracic region, dorsal and ventral to the shoulder blade, in areas most commonly struck during predatory episodes [45]. The control (non-envenomated) mouse was injected in the same regions with 100 μL of deionized water. Trials (10-minute trial duration) started as soon as the test apparatus was placed in the cage, with observers counting tongue flicks directed within 1 cm of either the envenomated or the non-envenomated mouse. All tongue flicking was recorded double blind to the condition; therefore, the observer was unaware of which mouse carcass was injected with the control or venom sample, as well as which condition was being tested. Tongue flicking in snakes represents a stimulus-seeking behavior that is the main process for delivering volatile and non-volatile cues to the vomeronasal organs [11]. Since tongue flicking is activated by the detection of volatile cues by the nasal olfactory system, or visual, thermal or vibratory stimuli, measuring the rate of tongue flicking is an accurate and convenient assay of nasal as well as vomeronasal chemoreception in snakes [11,46]. Cages and test apparatus were cleaned between trials.

### Experiment 2. Low-pressure size exclusion chromatography

Lyophilized venom (250 mg, from the same venom pool used in Experiment 1) was dissolved in 1.0 mL HEPES buffer solution (10 mM, pH 6.8, with 60 mM NaCl and 5 mM CaCl_2_) and briefly centrifuged at 9,000 rpm to pellet and remove insoluble material. This solution was then fractionated by size exclusion chromatography using a 90 × 2.8 cm column of BioGel P-100 equilibrated with the same HEPES buffer. Fractionation occurred at a flow rate of 6.3 mL/hr at 4°C, and 30-minute fractions were collected. Elution of size-fractionated protein and peptide peaks was monitored at 280 nm.

### Enzyme assays of fractionated venom

All BioGel fractions (10 μL/assay, in duplicate) were assayed for several enzymes common to most rattlesnake venoms [21], including exonuclease (phosphodiesterase), L-amino acid oxidase, caseinolytic metalloproteinase, thrombin-like and kallikrein-like serine proteinases and phospholipase A_2_, as described previously [47].

### Behavior trials using fractionated venom

Fractions of Peaks I to III were pooled separately, dialyzed in a 14 kDa cutoff membrane tubing (Peak I) or in a 3.5 kDa cutoff membrane tubing (Peaks IIa, IIb and III) against 2 × 2 liters of ddH_2_O, lyophilized and stored frozen at -20°C until use. Similar to Experiment 1, the experimental ("envenomated") mouse was injected with one of the four fractionated protein peaks (1.25 mg protein in 100 μL, reconstituted in ddH_2_O) or the combined peptide peaks (1.5 mg in 100 μL), and a non-envenomated control was injected with 100 μL ddH_2_O. When testing with fractionated venom, the number of subjects was limited by the quantity of protein in each peak. To induce SICS, each snake struck a mouse suspended by forceps just prior to placement of the apparatus; again, this mouse was immediately removed and discarded, never having touched the floor or walls of the cage. Trials began when the test apparatus containing E and NE carcasses was placed into the cage, again with 10-minute trials.

The mean number of tongue flicks directed towards the E and NE mouse carcasses for whole crude venom and each peak were compared using a two-sample t-test and Chi-square analysis (χ^2^). For all trials, the numbers of tongue flicks were converted to percentages (that is, percent tongue flicks emitted to E and NE mice) by dividing the number of tongue flicks aimed at the E carcass by the total number of tongue flicks for both carcasses. These data were analyzed by single sample *t*-tests in which mean percent tongue flicks directed toward envenomated mice were compared to 50%, the expected value under the null hypothesis. Rate of tongue flicking can be highly variable among snakes, so converting rate of tongue flicking to percentages places all snakes on the same scale. In addition, to achieve homogeneity of variance among conditions, we used a Log_10 _transformation to normalize data, which was analyzed by analyses of variance (ANOVA) followed by Newman-Keuls range test.

### Mass determination by mass spectrometry (MALDI-TOF-TOF)

Peak III from size exclusion (BioGel P-100 column) was desalted using C_4 _ZipTips (Millipore Inc., Billerica, MA, USA) and analyzed using a Bruker Ultraflex MALDI-TOF mass spectrometer (Proteomics and Metabolomics Facility, Colorado State University, Fort Collins, CO, USA) operating in linear mode. Protein (approximately 0.5 μg) was spotted onto a sinapinic acid matrix (10 mg/mL 50% acetonitrile, 0.1% trifluoroacetic acid; 1.0 μL) and spectra were acquired in the mass range of 3.0 to 25 kDa.

### Purification by reverse-phase high performance liquid chromatography (RP-HPLC)

Peak III was then further fractionated by reverse-phase high pressure liquid chromatography. Two hundred microliters (1.0 mg/mL) were injected onto a Grace Vydac Reverse Phase C_18 _(4.6 × 250 mm) column equilibrated with buffer A (0.1% Trifluoroacetic acid (TFA) in water). Absorbance was measured at 280 nm and proteins were eluted using a shallow gradient of 20% to 28% buffer B (80% acetonitrile in 0.1% TFA) over 50 minutes, with a flow rate of 1.0 ml/min. Peaks eluting at approximately 23% buffer B (fraction 13 - major peak; fraction 14 - minor following peak) were collected, dried in a Savant speedvac (ThermoScientific, Rockford, IL, USA), and stored at -20°C. Masses of proteins in fractions 13 and 14 were determined using a Bruker Ultraflex MALDI-TOF mass spectrometer (Bruker Corporation, Fremont, CA, USA) as above.

### N-terminal sequencing of reversed-phase HPLC purified proteins

Samples of Peak III for sequencing were reduced with dithiothreitol and alkylated with iodoacetamide as described previously [48]. The first 30 resides of sequence were obtained using an ABI Procise sequencer (Life Technologies/Applied Biosystems, Grand Island, NY, USA), and sequence obtained was subjected to Basic Local Alignment Search Tool (BLAST) at the National Center for Biotechnology Information (Bethesda, MD, USA) [49].

## Abbreviations

E: Envenomated; HPLC: high-pressure liquid chromatography; MALDI-TOF: Matrix-assisted laser desorption/ionization time-of-flight; NE: Non-envenomated; RGD: Arg-Gly-Asp acid; SICS: strike-induced chemosensory searching; SVMP: Snake venom metalloproteinase; TFA: Trifluoroacetic acid; TTX: tetrodotoxin

## Competing interests

The authors declare that they have no competing interests.

## Authors' contributions

AJS, DC and SPM conceived the study, planned experiments, and wrote and revised the manuscript. CB and AJS conducted behavioral trials. SPM conducted purification and characterization of venom proteins, mass spectometry and sequencing experiments. AJS and SPM conducted replications of purification of venom disintegrins. All authors have read and approved the manuscript.

## Supplementary Material

Additional file 1 **Table S1. Raw data: Number of tongue flicks toward envenomated (E) or non-envenomated (NE) mice**. This table contains the raw data collected for behavioral experiments 1 and 2. Experiment 1 consisted of paired trials using a non-envenomated vs. and envenomated (whole venom) mouse - this trial was conducted to replicate and confirm past results. Experiment 2 consisted of the same paired trials, but instead of whole venom, one of five size exclusion venom fractions, Peak I, IIa IIb, III or Peptides, was used in "envenomated" mice. Trials were of 10 minutes duration, and the number of tongue flicks directed toward one or the other mouse was recorded. Click here for file

Additional file 2 **Figure S1. Reducing SDS-PAGE analysis of size exclusion chromatography fractions**. Ten micrograms of protein (reduced with DTT) from each size exclusion peak (BioGel P100) were loaded onto a 12% acrylamide NuPage gel. Following electrophoresis, the gel was fixed and stained with 0.1% Coomassie Brilliant Blue R250 using standard methods, destained and photographed. MW standards = Invitrogen Mark 12. Circled faint bands indicate carryover contamination of metalloproteinases (darkest bands) from lanes 2 and 4, respectively. Note that lane 5 is the only peak containing disintegrin bands (dark pair, red bracket); peptides were not visualized and are smaller than the resolution capability of the gel. Click here for file
