# Supplementary material for: Molecular basis for prey relocation in viperid snakes
Source: BMC Biol. 2013 Mar 1;11:20. doi: 10.1186/1741-7007-11-20 (PMC3635877; doi:10.1186/1741-7007-11-20)
Supplement: Additional file 1 — Table S1. Raw data: Number of tongue flicks toward envenomated (E) or non-envenomated (NE) mice. This table contains the raw data collected for behavioral experiments 1 and 2. Experiment 1 consisted of paired trials using a non-envenomated vs. and envenomated (whole venom) mouse - this trial was conducted to replicate and confirm past results. Experiment 2 consisted of the same paired trials, but instead of whole venom, one of five size exclusion venom fractions, Peak I, IIa IIb, III or Peptides, was used in "envenomated" mice. Trials were of 10 minutes duration, and the number of tongue flicks directed toward one or the other mouse was recorded. [file 1741-7007-11-20-S1.DOCX]

**Supplemental Table 1. Raw data: Number of tongue flicks toward envenomated (E) or non-envenomated (NE) mice**

**Experiment 1 - Whole Venom**

**Subject E mouse NE mouse Proportion to Experimental**

1 46 50 .48

3 119 33 .78

4 129 71 .65

5 86 16 .84

6 17 13 .57

7 66 9 .88

8 119 35 .77

**Experiment 2 – Peak I**

**Subject E mouse NE mouse Proportion to Experimental**

1 32 61 .34

2 138 1 .99

3 129 175 .42

5 107 138 .44

6 59 115 .34

7 204 89 .70

1 53 69 .43

6 41 20 .67

1 56 48 .54

3 40 20 .67

2 0 11 .00

8 96 72 .57

8 37 114 .25

1 66 108 .38

1 24 45 .35

8 53 27 .66

8 33 33 .50

**Experiment 2 – Peak IIa**

**Subject E mouse NE mouse Proportion to Experimental**

1 67 56 .54

8 31 183 .14

3 30 36 .45

4 39 116 .25

5 25 30 .45

6 11 0 1.0

7 63 126 .33

3 122 63 .66

3 69 86 .45

1 148 64 .70

3 45 42 .52

**Experiment 2 – Peak IIb**

**Subject E mouse NE mouse Proportion to Experimental**

1 47 51 .48

5 32 32 .50

7 38 21 .64

8 56 28 .67

1 189 71 .73

3 22 17 .56

2 64 92 .41

1 98 124 .44

8 91 204 .31

3 55 93 .37

**Experiment 2 - Peak III**

**Subject E mouse NE mouse Proportion to Experimental**

1 89 20 .82

2 40 18 .69

3 51 51 .50

4 67 52 .56

5 37 11 .77

6 105 48 .69

7 14 12 .54

8 48 13 .79

3 33 20 .62

1 51 27 .65

9 55 7 .89

**Experiment 2 – Peptide Peaks**

**Subject E mouse NE mouse Proportion to Experimental**

1 120 82 .59

2 46 25 .65

3 15 2 .88

6 23 24 .49

4 7 39 .15

5 9 19 .32

1 30 34 .47

6 225 19 .92

2 0 53 .00
