# Supplementary figures and images for: Molecular basis for prey relocation in viperid snakes
Source: BMC Biol. 2013 Mar 1;11:20. doi: 10.1186/1741-7007-11-20 (PMC3635877; doi:10.1186/1741-7007-11-20)

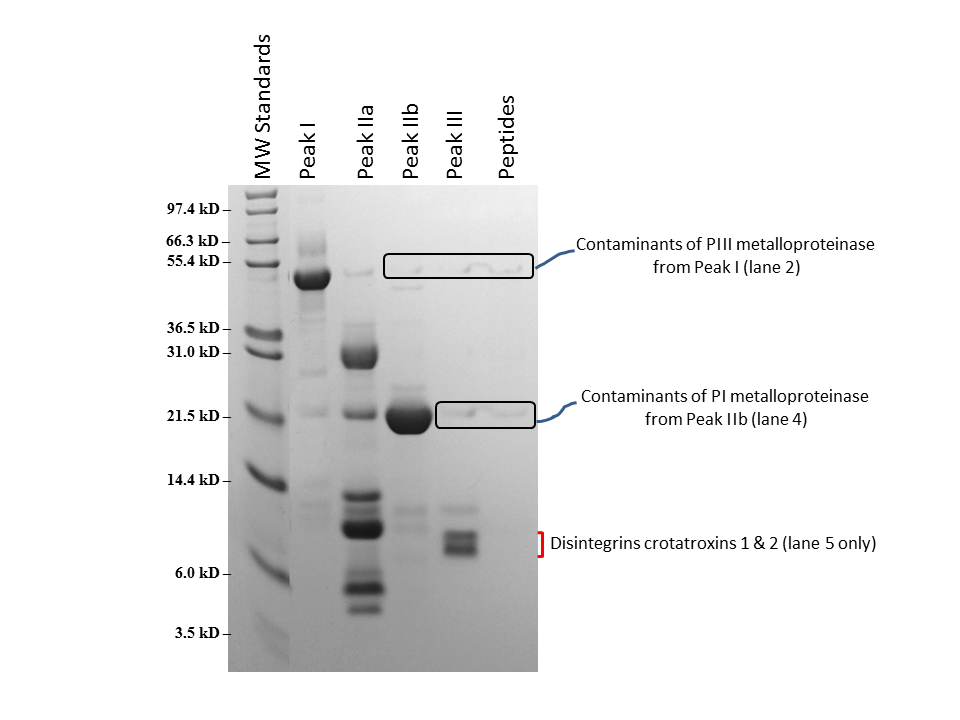

Supplement: Additional file 2 — Figure S1. Reducing SDS-PAGE analysis of size exclusion chromatography fractions. Ten micrograms of protein (reduced with DTT) from each size exclusion peak (BioGel P100) were loaded onto a 12% acrylamide NuPage gel. Following electrophoresis, the gel was fixed and stained with 0.1% Coomassie Brilliant Blue R250 using standard methods, destained and photographed. MW standards = Invitrogen Mark 12. Circled faint bands indicate carryover contamination of metalloproteinases (darkest bands) from lanes 2 and 4, respectively. Note that lane 5 is the only peak containing disintegrin bands (dark pair, red bracket); peptides were not visualized and are smaller than the resolution capability of the gel. [file 1741-7007-11-20-S2.TIFF]
